# Centralized Networks to Generate Human Body Motions

**DOI:** 10.3390/s17122907

**Published:** 2017-12-14

**Authors:** Sergei Vakulenko, Ovidiu Radulescu, Ivan Morozov, Andres Weber

**Affiliations:** 1 Institute for Mechanical Engineering Problems, 195251 Saint Petersburg, Russia; vakulenfr@mail.ru; 2 Mechanics and Optics, Saint Petersburg National Research University of Information Technologies, 191119 Saint Petersburg, Russia; 3 DIMNP-UMR 5235 CNRS/UM, University of Montpellier, 34095 Montpellier, France; ovidiu.radulescu@umontpellier.fr; 4 Computer Science Department, University of Bonn, 53113 Bonn, Germany; mmmmoroz@yandex.ru

**Keywords:** neural networks, markers, human body motions, motion sensors, motion representation, motion reconstruction

## Abstract

We consider continuous-time recurrent neural networks as dynamical models for the simulation of human body motions. These networks consist of a few centers and many satellites connected to them. The centers evolve in time as periodical oscillators with different frequencies. The center states define the satellite neurons’ states by a radial basis function (RBF) network. To simulate different motions, we adjust the parameters of the RBF networks. Our network includes a switching module that allows for turning from one motion to another. Simulations show that this model allows us to simulate complicated motions consisting of many different dynamical primitives. We also use the model for learning human body motion from markers’ trajectories. We find that center frequencies can be learned from a small number of markers and can be transferred to other markers, such that our technique seems to be capable of correcting for missing information resulting from sparse control marker settings.

## 1. Introduction

In recent years, various neural network topologies have been used for recognizing and representing human body motions. In particular, the use of deep networks has been proposed [1,2] or of Long Short-Term Memory (LSTM) networks and their extensions [1,3,4]. Additionally, specialized architectures for human motions such as so-called “phase-functional networks” [5] have recently been proposed.

In this paper we advocate the use of another kind of network—the so-called centralized network [6]. Inspired by the success of these networks in neuroscience, genetics, and ecology [7,8,9,10,11,12,13], we consider centralized, continuous-time recurrent networks of an analogous topological structure as dynamical models for the simulation of human body motions.

Our method combines nonlinear oscillators, centralized architectures, and approximation by radial basis functions. All these ingredients are present in different fields in neuroscience, robotics, and machine learning, but to the best of our knowledge they have not yet been put together. Nonlinear oscillators were discovered as building blocks of locomotor neural circuits in animals, and similar designs were mimicked to control the movements of robots [14,15,16]. Although the idea of coupling oscillators to neural networks was successfully used to model gait transitions in cybernetic models [15,17], there is no systematic approach for learning complicated body movements from sensors data based on this idea. In order to do so, we use radial basis function networks—a popular general-purpose approximation method used in signal processing and system identification [18,19]. This machine learning technique has already been used in the context of locomotion, but for models different from ours [20]. The interest of our centralized architecture relating a few pacemaker hubs to satellite effectors is manifold. Beyond realism, it allows a robust control of body motions. The learning of the model can be decomposed into two parts. The first part is estimation of the pacemaker frequencies, which can be done using the signal from any effector or from a small group of randomly chosen effectors. The second part is also robust, being based on simple, two-layer, feed-forward radial basis function networks. Several extensions of this basic approach imply recursive networks. One of these extensions, based on a switching module with feedback, is discussed in the paper. Other extensions could consider couplings between oscillating nodes, directly or via satellite nodes. The interest in coupling lies in the possibility of synchronization, which has been shown to characterize gaits defined as collective nonlinear modes in the entire body [15,17].

Although to the best of our knowledge centralized networks have not directly been used in the context of human body motions, the widely considered *dynamical movement primitives* (DMPs) [21,22,23,24,25] share common grounds on a technical basis in several respects.

Our proposal not only relates the concept of DMPs to neural networks, but also generalizes and enhances their construction—for example, by transparently allowing more than one central oscillator and also incorporating switching while staying in the topological realm of centralized networks.

Our model allows us to sufficiently simulate long motions by only two oscillators. However, in some difficult cases (for example, if a motion consists of walking, running, kicking, punching, and knee kicking), we also decompose the whole motion into 2–4 segments and then for each segment we adjust the corresponding oscillator frequency. Then, the application of our approximation algorithm allows us to automatically obtain a uniform and smooth approximation of the whole motion. The use of 2–3 oscillators and 20–200 satellites has proven to be sufficient in our experiments.

Due to the network switching module we can use nonlinear oscillators and obtain a global network that can simulate a large class of different motions.

Our approach is trajectory-based, and works in principle on single marker trajectories independently of others. In this respect, our approach is similar to the DMP, but is in contrast to the Bayesian approaches presented in the literature, in which prior information has to be collected on the level of pose similarity using correlations of the positions of different body parts [26,27,28]. Our approach can be applied on the basis of a single motion and single trajectory, not requiring a database of collected motions as a priori knowledge.

The paper is organized as follows: In Section 2.1, we first give background on scale-free networks and centralized networks in Section 2.1.1. We define the centralized networks for elementary human motion in Section 2.1.2, and describe our approach to change frequencies, hence building centralized networks generating a large class of human body motions in Section 2.1.3. The idea of a switching module is detailed in Section 2.2. Algorithms to construct such networks to generate human body motions are given in Section 2.3—first for the case of non-segmented motions in Section 2.3.1, and then for the case of segmented motions in Section 2.3.2. A comparison of our approach with the DMPs is given in Section 2.4. Experimental results are presented in Section 3. In Section 4, we not only discuss the results in relation to previous and state-of-the-art methods, but we also give some directions for possible future works.

## 2. Materials and Methods

### 2.1. Network Structure

#### 2.1.1. Background on Scale-Free Networks and Centralized Networks

Networks of dynamically coupled elements have imposed themselves as models of complex systems in physics, chemistry, biology, and engineering. An important structure-related property of networks is their scale-freeness [8,9,29,30], often invoked as a paradigm of self-organization, and the spontaneous emergence of complex collective behavior. In scale-free networks, the fraction P(k) of nodes in the network having *k* connections to other nodes (i.e., having degree *k*) can be estimated for large values of *k* as $P\left(k\right)\;∼\;k^{-\gamma }$, where γ is a parameter whose value is typically in the range 2<γ<3 [29]. In such networks, the degree is extremely heterogeneous. In particular, there are strongly connected nodes that can be named hubs, or centers. The hubs communicate to each other directly, or via a number of weakly connected nodes. The weakly connected nodes that interact mainly with hubs can be called satellites. Scale-free networks also have nodes of intermediate connectivity. Networks that have only two types of nodes—strongly connected hubs and weakly connected satellites—are known as bimodal degree networks [31]. Because of the presence of a large number of hubs, scale-free or bimodal degree networks can be called centralized.

It has been shown that centralized networks show a good compromise between robustness and flexibility. They are resilient with respect to external perturbations and are insensitive to noise, while remaining totally controllable [32,33,34]. Furthermore, centralized networks are universal approximate models, and can simulate any structurally stable dynamics [6,35,36]. Other interesting dynamical properties of centralized networks are related to their ability to switch, activating on turning the coordinated evolution of different sets of nodes. On one hand, this capacity is responsible for the “stable yet switchable” property, meaning that the network remains stable in a given context and is able to reach another stable state when a stimulus indicates a change in the context [6]. On the other hand, centralized networks can be itinerant; i.e., spontaneously changing their functioning mode [10].

The above dynamical properties of centralized networks have received particular attention in neuroscience, genetics, and ecology. Centralized connectivity has been found by functional imaging of brain activity in neuroscience [7], and also by large-scale studies of protein–protein interactions or of metabolic networks in functional genetics [8,9]. Itinerant and switching behavior was observed in the transient activity of antennal lobe neurons involved in insect olfaction or in the activity of high vocal centers controlling songbird patterns [10]. The robustness of scale-free networks was emphasized in relation to food-webs and ecosystems [11,12], epidemics [13], etc.

Motivated by the success of centralized networks in neuroscience, genetics, and ecology, we consider centralized, continuous-time recurrent networks of an analogous topological structure as dynamical models for simulation of human body motions. From the general setting, we take the idea that these networks consist of a few centers and many satellites. As human motions are very often cyclical but with varying frequencies, the centers may evolve in time as oscillators with different frequencies. We take the idea of radial basis function (RBF) networks to define the center states. An additional switching module allows us to turn from one particular motion to another. Due to this structure, the network can simulate a large class of different motions with good accuracies, which depend on the oscillator frequencies.

#### 2.1.2. Centralized Networks for Elementary Human Motions

The networks consist of *n* centers with the states $q_{i}$, and a number of satellites with states $X_{j},Y_{j},Z_{j}$, where j=1,…,N≫n. In the simplest case, when we approximate a single relatively simple motion, the time evolutions of the center states are governed by harmonic oscillator equation:

(1) $$\frac{d^{2}q_{i}}{dt^{2}}+\omega_{i}^{2}q_{i}=0,\;i=1,\ldots ,n,$$

where $q_{i}$ is the coordinate of the *i*-th oscillator, $\omega_{i}$ is the frequency of that oscillator, and *n* is the number of oscillators. Often even two oscillators (n=2) provide a good accuracy, but for more complicated motions one can take n∈{3,4,5}. Let $q\left(t\right)=\left(q_{1},\ldots ,q_{n}\right)$ be the vector of the oscillator states, depending on time *t*, and $x_{k}\left(t\right)$ are output coordinates (here $x_{1}\left(t\right)=X\left(t\right),x_{2}\left(T\right)=Y\left(t\right),x_{3}\left(t\right)=Z\left(t\right)$).

The centers are connected with *N* output coordinates $x_{k}$ by a network:

(2) $$x_{k}=\sum_{j=1}^{N_{m}}W_{kj}\Phi_{j}\left(q,b\right)\;,$$

where $x_{k}$ is the *k*-th coordinate on the body, k=1,…,N. The functions $\Phi_{j}$ form a basis in the space $L_{2}(\left[-X_{0},X_{0}\right]$, where $x_{0}$ is characteristic maximal amplitude of motion for the *j*-th point, *b* is a parameter, and $N_{m}$ is the number of basis functions. The matrix entry $W_{kj}$ describes the action of the node *j* on $x_{k}$. Note that (2) defines a straight-forward network that maps the center states $q_{i}$ into the output coordinate $x_{k}$ by $N_{m}$ hidden neurons (satellites), and therefore, there are no interactions between satellites.

There are possible different choices of $\Phi_{j}$. For example, we can consider the following cases.

**A**  Harmonic basis. Here we assume that
  (3) $$\Phi_{j}\left(q,b\right)=\cos\left(bjq\right),$$
  where *b* is a frequency.
**B**  System of radial basis functions.
  For the case where a motion consists of many segments and we observe sharp transitions between those segments, we can use radial basis functions
  (4) $$\Phi_{j}=\phi (b|q-{\bar{q}}^{\left(j\right)}\left|\right),\;j=1,\ldots ,N_{m},$$
  where ϕ is a fixed function, *b* is a sharpness parameter, and ${\bar{q}}^{\left(j\right)}$ is the vector of centers of radial basis functions with components ${\bar{q}}^{(j})=\left({\bar{q}}_{1}^{\left(j\right)},\ldots ,{\bar{q}}_{n}^{\left(j\right)}\right)$, where the latter are parameters of the system, and |z| denotes the Euclidian norm of the vector *z*: $\left|z\right|=\sqrt{\sum_{i=1}^{n}z_{i}^{2}}$. We assume that the radial basis function ϕ(|z|) is well localized at z=0 and is smooth. For example, we can take the Gaussian
  (5) $$\phi \left(|z|\right)={\exp(-|z|}^{2}/2).$$
**C**  Polynomial basis.
  Here we take
  (6) $$\Phi_{j}\left(q\right)=q^{j-1},\;j=1,\ldots ,N_{m}.$$
  The basis **B** has an important advantage: the radial basis functions provide local approximations that are important to approximate complicated motions with sharp transitions.
  To perform switching in the network, we will also use the sigmoidal functions σ. They are increasing and smooth (at least twice differentiable) functions such that
  (7) $$\sigma \left(-\infty \right)=0,\;\sigma \left(+\infty \right)=1,\;\sigma^{\prime }\left(z\right)>0.$$
  Typical examples can be given by
  (8) $$\sigma \left(h\right)=\frac{1}{1+\exp(-h)},\;\sigma \left(h\right)=\frac{1}{2}\left(\frac{h}{\sqrt{1+h^{2}}}+1\right).$$

**Figure 1:**
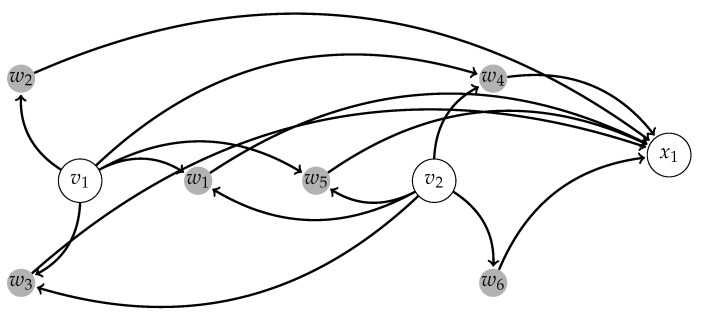
This image shows the control of one of the *x*-coordinates of human body motions by a network consisting of two oscillators $\left(v_{1},v_{2}\right)$ and a radial basis function (RBF) network with N=6 nodes. The graph consists of eight nodes denoted by $v_{1},v_{2},w_{1},w_{2},w_{3},w_{4},w_{5},w_{6}$. Each node $w_{i}$ corresponds to a contribution of a radial basis function $\Phi (q-{\bar{q}}^{\left(j\right)})$. The nodes $v_{1},v_{2}$ form the set of centers C and they affect $w_{i}$. In turn, the nodes $w_{i}$ determine the output coordinate $x_{1}$.

The structure of interactions between centers and coordinates $x_{i}$ can be described by Figure 1.

#### 2.1.3. Centralized Networks Generating a Large Class of Human Body Motions

To approximate different motions by a single network, we should have the possibility of changing the frequencies and coefficients $W_{kj}$.

The main idea is as follows. Each motion can be approximated by a network described in the previous subsection, with adjusted frequencies $\omega_{i}$ and appropriated coefficients $W_{kj}$. We can use nonlinear oscillators to obtain all possible frequencies. For example, one can use the model described below. Consider networks consisting of *n* centers, which evolve as nonlinear oscillators:

(9) $$\frac{d^{2}q_{i}}{dt^{2}}+z_{c}f\left(q_{i}\right)=0,\;i=1,\ldots ,n,$$

where $q_{i}$ is the coordinate of the *i*-th oscillator, f(q) is a nonlinear function, and $z_{c}$ is a control paremeter (one can take, for instance, f=sin(q) or $f=aq-bq^{3}$). We assume that

(10) $$q_{i}\left(0\right)=0,\;p_{i}\left(0\right)=p_{0},\;p\left(t\right)=\frac{dq}{dt},$$

where $p_{0}$ is a fixed number. Solutions of Equation (9) are periodic functions of time, with the period $T(z_{i})$ and the frequency $\omega (z_{i})=2\pi /T$. It can be found by the motion integral of Equation (9) that:

(11) $$E_{i}=\frac{1}{2}{\left(\frac{dq_{i}}{dt}\right)}^{2}+F\left(q_{i},z_{i}\right),$$

where *F* is the antiderivative of *f*: $f\left(q\right)=\frac{dF}{dq}$.

Notice that Equations (2)–(9) are “standard” (see e.g., [19]).

Consider a set of human motions characterized by a set of coordinates $x_{1}^{\left(j\right)},\ldots ,x_{N}^{\left(j\right)}$, where the upper index *j* corresponds to a particular motion. Each motion can be described by the model (1) and (2) with the corresponding frequencies $\omega_{i}^{\left(j\right)}$ and coefficients $W_{kl}^{\left(j\right)}$.

A switching between the different motions can be performed by a choice of the control parameters $z_{i}$.

By the switching module (described in the next subsection), we find a network subsystem which has $z_{c}^{\left(j\right)}$ as local attractors. Then, we can construct maps $z_{c}\to \omega_{1}\left(z\right),\ldots ,\omega_{n}\left(z\right)$ and $z_{c}\to W_{kl}\left(z_{c}\right)$ such that

(12) $$\omega_{l}^{\left(z_{l}^{\left(i\right)}\right)}=\omega \left(z_{l}^{\left(i\right)}\right),\;l=1,\ldots ,n$$

(13) $$W_{li}^{\left(j\right)}=W_{ki}\left(z^{\left(j\right)}\right).$$

Hence, our global model for human motion consists of

1. a system of *n* nonlinear oscillators (9) with the control parameters $z_{i},i=1,\ldots ,n$;
2. an RBF network defined by (2);
3. maps obeying Equations (12) and (13); and
4. a switching module that is a network with M+1 nodes, where *M* is the number of different motions.

In the next section, we describe the switching module.

### 2.2. Switching Module

**Figure 2:**
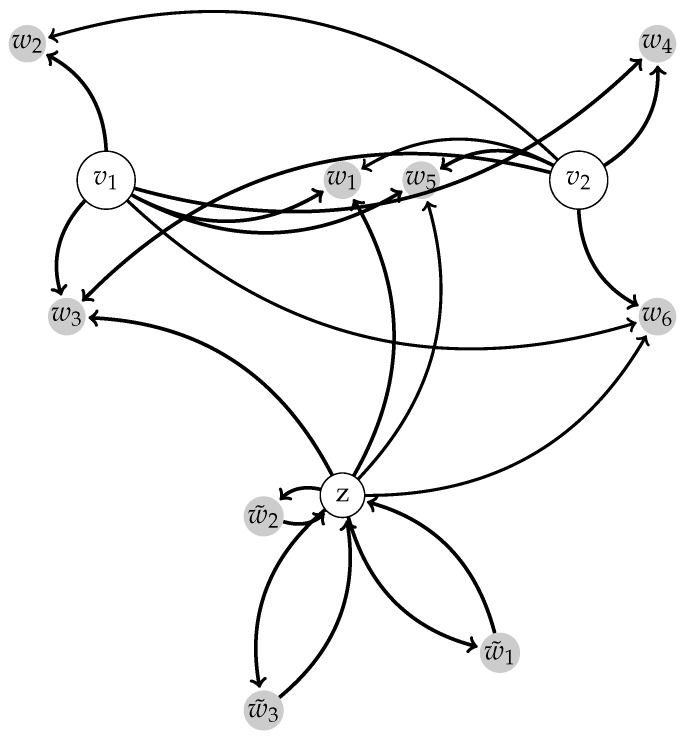
Modular architecture. This can be seen as an example of the architecture described in [6]. The switching module consists of the center *z* and the satellites ${\tilde{w}}_{1},{\tilde{w}}_{2},{\tilde{w}}_{3}$. The generating module consists of the centers $v_{1},v_{2}$ and the satellites $w_{1},\ldots ,w_{6}$. Note that there is a feedback between *z* and the satellites $w_{i}$; however, there is no feedback of $w_{j}$ on $v_{l}$.

*Ideas behind construction*. Before stating a formal statement, we present a brief outline which describes the main ideas of the proof and the architecture of the switchable network. The network consists of two modules. The first module is a generating one and it is a centralized neural network with *n* centers $q_{1},\ldots ,q_{n}$ and satellites $x_{1},\ldots ,x_{N}$. The second module consists of a center $v_{n+1}=z$ and *m* satellites ${\tilde{w}}_{1},\ldots ,{\tilde{w}}_{m}$. The satellites from this module interact only with the module center *z*; i.e., in this module the interactions can be described by a distar graph [6]. Only the center of the second module interacts with the neurons of the first (generating) module. We refer to the second module as a switching one. This architecture is shown in Figure 2.

For the switching module, the corresponding differential equations have the following form. Let us consider a distar interaction motif, where a node *z* is connected in both directions with *m* nodes ${\tilde{w}}_{1},\ldots ,{\tilde{w}}_{m}$. By this notation, the equations for the switching module can be written down in the form

(14) $$\frac{d{\tilde{w}}_{i}}{dt}=\sigma \left({\tilde{b}}_{i}z-{\tilde{h}}_{i}\right)-\kappa^{-1}{\tilde{w}}_{i},$$

(15) $$\frac{dz}{dt}=\sigma \left(\kappa^{-1}\sum_{j=1}^{m}{\tilde{a}}_{j}{\tilde{w}}_{j}-h\right)-\xi \bar{\lambda }z,$$

where i=1,…,m and ${\tilde{b}}_{i},{\tilde{a}}_{j},\bar{\lambda }>0$.

In order to come up with a mathematical description of the way in which switching module works, let us consider the system of differential equations

(16) $$\frac{dv}{dt}=Q\left(v,z\right),\;v=\left(v_{1},\ldots ,v_{2n}\right)\;,$$

where *z* is a real control parameter. Let $z_{1},\ldots ,z_{m+1}$ be some values of this parameter. We find a vector field *Q* such that for $z=z_{l}$, where l=1,…,m, the dynamics defined by (16) have the prescribed dynamics. For example, we can set n=2 and

(17) $$v_{1}=q,\;v_{2}=p=\frac{dq}{dt},$$

and

(18) $$Q=v_{2},\;\frac{dv_{2}}{dt}=z_{c}f\left(v_{1}\right)\;,$$

which gives (9).

For the switching module, we adjust the center–satellite interactions and the center response time parameter ξ in such a way that for a set of values ξ the switching module has the dynamics of the system shown in (14) and (15), with *m* different rest points $z=z_{1},z_{2},\ldots ,z_{m+1}$, and for sufficiently large ξ the system shown in (14) and (15) has a single equilibrium close to $z_{1}=0$. The existence of such a choice will be shown in Lemma 1. This lemma has been stated and proven in the generic context ([6] Lemma 8.2). Due to its importance, we restate it here.

**Lemma** **1.** *Let β∈(0,1) and let m be a positive integer. For sufficiently small κ>0, there exist ${\bar{a}}_{j},b_{i},{\tilde{h}}_{i},h$ such that*

*(***i***)*  *for an open interval of values ξ the system in (14) and (15) has m stable hyperbolic rest points*
  (19) $$z_{j}\in \left(j-1+\beta ,j+\beta \right),$$
  *where j=1,…,m;*
*(***ii***)*  for $\xi >\xi_{0}>0$ the system in (14) and (15) has a single stable hyperbolic rest point.

For the proof of this lemma we refer to [6].

### 2.3. Algorithm of Construction of the RBF Network to Generate Human Body Motions

#### 2.3.1. Non-Segmented Motions

Simple motions can be handled as a whole (i.e., without any segmentation). Let us fix the index *j* (i.e., consider a particular motion). Let $t_{1},\ldots ,t_{K}$ be time moments where we have data on human body coordinates $X_{j}\left(t\right),Y_{j}\left(t\right),Z_{j}\left(t\right)$, where *j* is the index of an optical marker on the body and the number of the markers is *N*, j=1,…,N. All X,Y, and *Z* are thus vectors with *N* components. Let ε(k,ω) be the $L_{2}$- approximation accuracy for the *x*-component and *k*-th marker defined by

(20) $$\varepsilon_{X}^{2}\left(k,\omega \right)=\sum_{m=1}^{K}{\left(X_{k}\left(t_{m}\right)-x_{k}\left(q\left(t_{m}\right),\omega \right)\right)}^{2},$$

where $x_{k}\left(q\right)$ are defined by (2). Similarly,

(21) $$\varepsilon_{Y}^{2}\left(k,\omega \right)=\sum_{m=1}^{K}{\left(Y_{k}\left(t_{m}\right)-y_{k}\left(q\left(t_{m}\right),\omega \right)\right)}^{2},$$

(22) $$\varepsilon_{Z}^{2}\left(k,\omega \right)=\sum_{m=1}^{K}{\left(Z_{k}\left(t_{m}\right)-z_{k}\left(q\left(t_{m}\right),\omega \right)\right)}^{2}\;.$$

The relative accuracies for X,Y,Z components are given by

(23) $$\varepsilon_{r,X,\omega }^{2}\left(k\right)=\varepsilon_{X}^{2}\left(k,\omega \right)/\sum_{m=1}^{K}X_{k}{\left(t_{m}\right)}^{2},$$

(24) $$\varepsilon_{r,Y,\omega }^{2}\left(k\right)=\varepsilon_{Y}^{2}\left(k,\omega \right)/\sum_{m=1}^{K}Y_{k}{\left(t_{m}\right)}^{2},$$

(25) $$\varepsilon_{r,Z,\omega }^{2}\left(k\right)=\varepsilon_{Z}^{2}\left(k,\omega \right)/\sum_{m=1}^{K}Z_{k}{\left(t_{m}\right)}^{2},$$

respectively. Let us fix a k∈{1,2,…,N} (i.e., a marker on the human body). For a set of frequency vectors ω, we compute the integral relative accuracy

(26) $$\varepsilon_{r,k}\left(\omega \right)=\sqrt{(\varepsilon_{r,X,\omega }^{2}\left(k\right)+\varepsilon_{r,Y,\omega }^{2}\left(k\right)+\varepsilon_{r,Z,\omega }^{2}\left(k\right))/3}.$$

Then, we find a $\omega^{*}$ such that $\varepsilon (\omega_{*})$ is minimal:

(27) $$\omega_{*}=\mathrm{argmin}\;\varepsilon_{r,k}\left(\omega \right).$$

The corresponding coefficients $W_{kl}$ can be found by the standard Matlab programs, which approximate a target function by RBF networks. Here we use standard radial basis functions of Gaussian type, where the sharpness parameter *b* can be adjusted by trial and error to minimize ε.

Numerical results show that the frequencies found for a particular motion by a value of *k* (a specific marker choice) and giving a small $\varepsilon_{r,k}$ can be applied to find good approximations for all rest values of *k* (i.e., for all other markers). An alternative method is to take the average of all markers, and then

$$\omega_{*}=\mathrm{argmin}\;\sum_{k}\varepsilon_{r,k}\left(\omega \right).$$

However, in this case the running time of the algorithm sharply increases.

#### 2.3.2. Segmented Motions

For complex motions it is difficult to uniformly approximate a whole motion using a few neurons; sometimes such approximation is good anywhere except for a certain interval. In fact, it is difficult to expect that all parts of complicated motions consisting of quite different elementary submotions can be handled with the same frequencies. However, we can use the segmentation. We then decompose the motion in segments $\left[T_{i},T_{i+1}\right]$, where $i=1,\ldots ,N_{\mathrm{seg}}$. For each segment we can determine optimal frequencies as described above and compute the accuracies. The frequency optimization can be done in two ways. If the number of oscillators is small (say, n=1,2), we can perform an exhaustive search over a uniform grid. For larger *n*, one can use a random search.

### 2.4. Comparison with DMPs

Let us compare the approach based on centralized networks, proposed in this present paper, and the classical method of dynamic movement primitives (DMPs). Both approaches use the same general representation, which, following [24], we write down as follows (see Equations (1) and (2) in [24]):

(28) $$\begin{array}{ccc} \frac{ds}{dt} & = & \mathrm{Canonical}(t,s), \end{array}$$

(29) $$\begin{array}{ccc} \frac{dy}{dt} & = & \mathrm{Transform}(t,y)+\mathrm{Perturbation}(s). \end{array}$$

The first equation is a time-dependent dynamical system, and the second one describes a transformation of trajectories of that dynamical system to desired trajectories y(t). Note that the term P(s)=Perturbation(s) should be adapted to induce a desired behaviour in the system; i.e., to reproduce a given trajectory [24]. So, a DMP consists of two parts, as described by Ernesti et al. [24]: “the canonical system and the transformation system. While the canonical system defines the state of the DMP in time, the transformation system is the link between this DMP state and the robot. The transformation system can be easily adapted to a desired trajectory; i.e., by solving a standard regression problem. The canonical system determines the type of attractor which can be either discrete or periodic”.

The DMP method uses P(s) to attain the twofold goal: to represent trajectories tending to rest points and periodic trajectories. In fact, roughly speaking, the dynamics of any dissipative systems reduce to some transient trajectories and motions on local attractors. However, it is not so simple to represent simultaneously transient dynamics, as was mentioned in [22]. To attain this goal, we must use sufficiently sophisticated formulas for P(s), which are based mainly on radial basis functions and the fact that RBFs are universal approximators.

In our centralized network approach, we use the same transformation system (29). However, we add a new idea in the representation of canonical part (28). It is well known that many motions generated by dissipative systems consist of slow and fast components. Fast components can describe, for example, transient trajectories, while slow components correspond to motions on local attractors. To represent such complex dynamics, we can nonetheless use systems of oscillators [37].

In particular, in our approach we usually use two oscillators, one of higher frequency and another of low frequency, although one can take three or more oscillators for complicated target motions. This idea works well: we greatly simplify the complicated formulas suggested in [22], and all transformation systems take the feed-forward form:

(30) y=Perturbation(s).

## 3. Results

For empiric tests we use the CMU Motion Capture Database [38]. We use two motions from family number 86, as these consist of sequences of several different motions performed by one actor subsequently, and hence have also been used as a test suite for different motion segmentation algorithms (e.g., [39] and references therein).

We use markers on left and right heels and left and right wrists, as in general from the position of these four markers even the full body motion can be reconstructed quite well [40,41].

### 3.1. Results without Segmentation and Ad Hoc Segmentations

We have considered two representative motions: Trial 1 and Trial 2. The first motion consists of jumping, hopping, turning, kicking, and punching, the second one is comprised of walking, squatting, running, stretching, jumping, punching, and drinking. The first motion is split into four segments [1,1300],[1300,2000],[2000,3000],[3000,4500], which were chosen visually by hand. Notice that the sampling rate for all examples was 120 Hz, so that the length of the motion segments are 10.8 s, 5.8 s, 8.3 s, and 12.5 s. The first segment consists of walking and hopping motions, the second one of a walking and a turning motion, the third one of punching (alternatively with both arms) and walking, and the fourth one of kicking (the right leg) and punching (alternatively with both arms). Similarly, the second motion was decomposed into segments [1,1800],[1800,2500],[2500,4500]. Hence, the lengths of the segments are 15.0 s, 5.8 s, and 16.7 s. The first segment consists of walking and squats, the second one of running (in a circle), and the third one of stretches.

An overview of results is given in Table 1.

**Figure 3:**
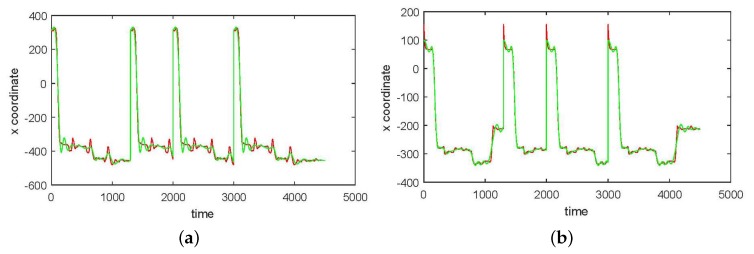

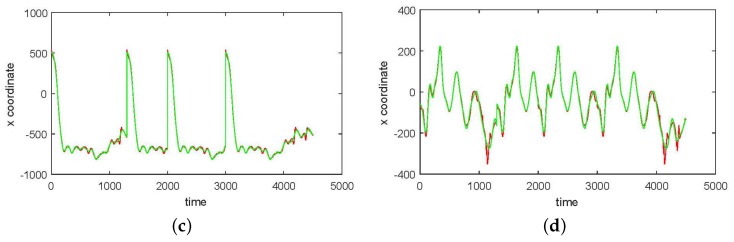
An approximation of *x*-coordinates by 25 satellites and two centers of motion (CMU 86 Trial 1) consisting of jumping, kicking, and punching (sampled at 120 Hz). (**a**) Right heel; (**b**) Left heel; (**c**) Right wrist, distal; (**d**) Left wrist, distal. The red curve shows the experimentally observed coordinates and the green curve gives their neural approximations.

**Figure 4:**
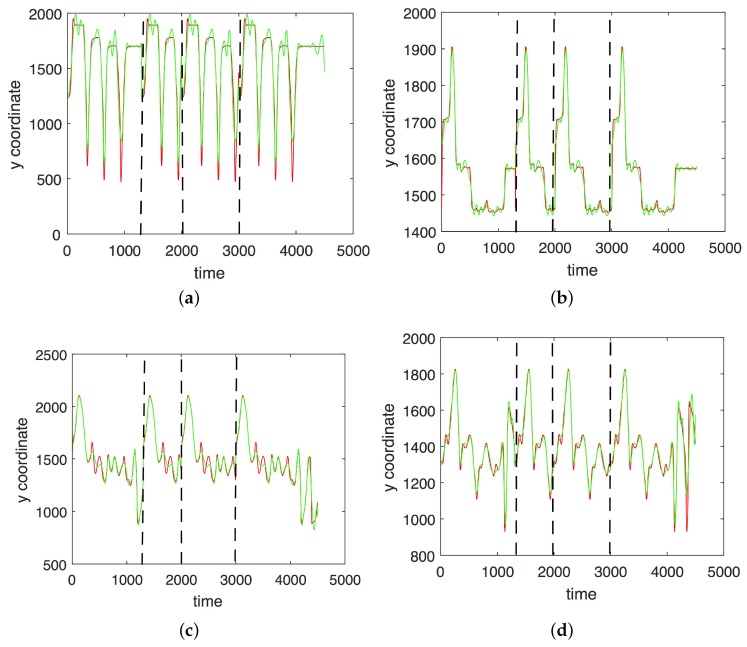
An approximation of *y*-coordinates by 25 satellites and two centers of motion (CMU 86 Trial 1; sampled at 120 Hz). (**a**) Right heel; (**b**) Left heel; (**c**) Right wrist, distal; (**d**) Left wrist, distal. The red curve shows the experimentally observed coordinates and the green curve gives their neural approximations.

**Figure 5:**
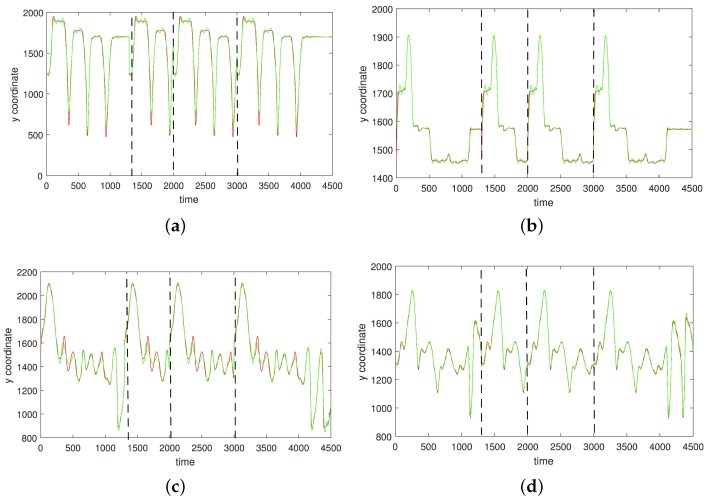
An approximation of *y*-coordinates by 50 satellites and two centers of motion (CMU 86 Trial 1; sampled at 120 Hz). (**a**) Right heel; (**b**) Left heel; (**c**) Right wrist, distal; (**d**) Left wrist, distal. The red curve shows the experimentally observed coordinates and the green curve gives their neural approximations.

**Figure 6:**
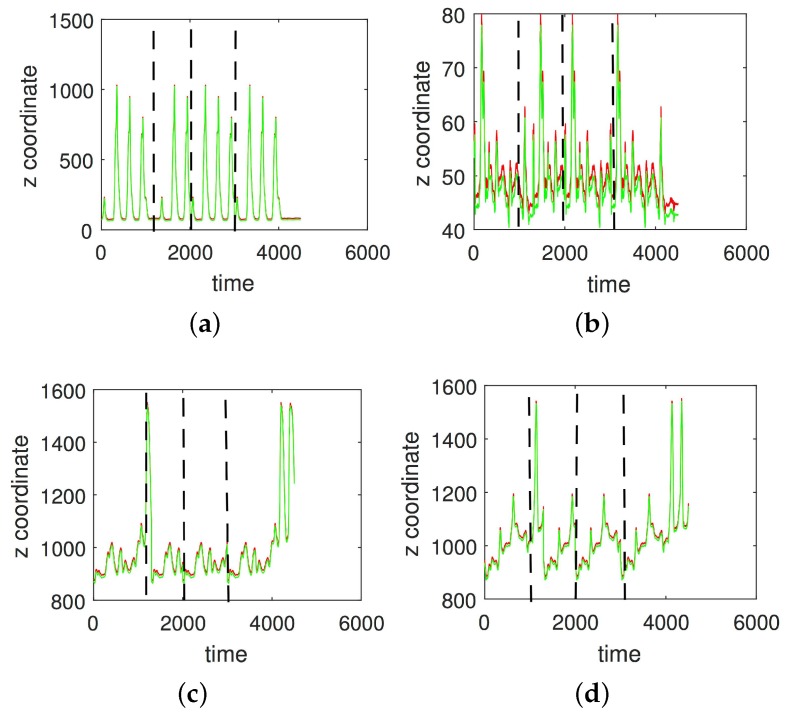
An approximation of *z*-coordinates by 100 satellites and two centers of motion (CMU 86 Trial 1; sampled at 120 Hz). (**a**) Right heel; (**b**) The left heel; (**c**) Right wrist, distal; (**d**) The left wrist, distal. The red curve shows the experimentally observed coordinates and the green curve gives their neural approximations.

**Figure 7:**
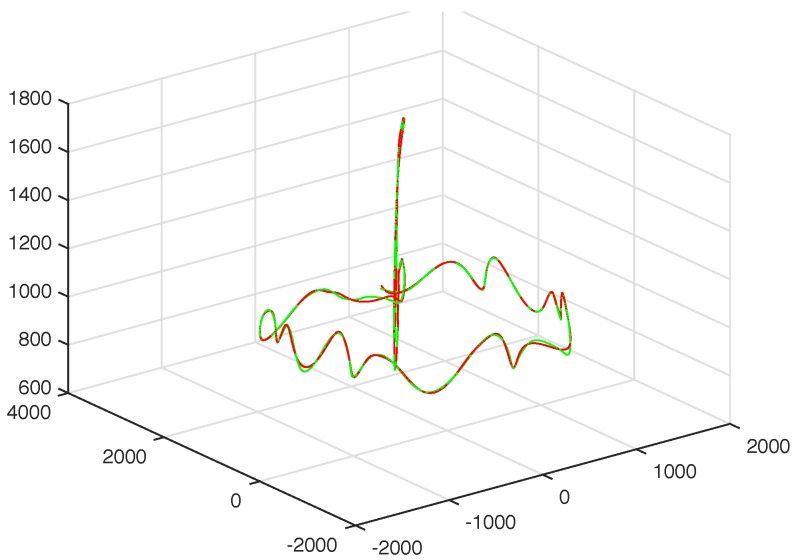
Three-dimensional plot of marker trajectory of the motion of the right wrist (distal; CMU 86 Trial 1). The red curve shows the experimentally observed coordinates and the green curve gives their neural approximations (with two centers and 100 satellites).

In Figure 3, Figure 4, Figure 5 and Figure 6 we show the results of the approximations of the different marker coordinates by 25, 50, and 100 satellites and two oscillators of motion CMU 86 Trial 1 consisting of jumping, kicking, and punching. In Figure 7 a three-dimensional plot of the marker trajectory of the right wrist of the same motion is presented.

**Figure 8:**
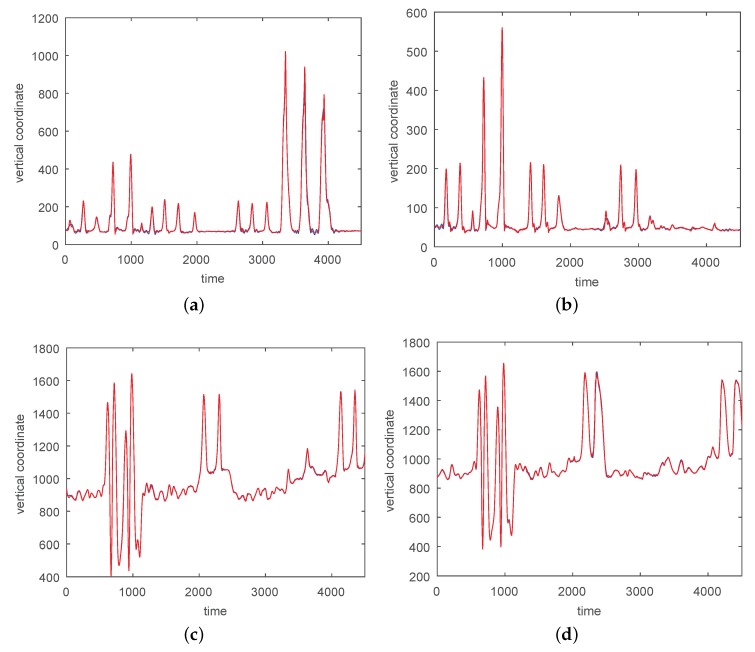
An approximation of *z* (vertical) coordinates by 200 neurons and two centers for a simple non-segmented motion (sampled at 120 Hz). The center frequencies are 0.30Hz and 0.72Hz. (**a**) *z*-coordinate for the right heel; (**b**) The left heel; (**c**) *z*-coordinate for the right wrist, distal; (**d**) The left wrist, distal. The red curves show the experimentally observed coordinates and the blue curves give their neural approximations.

**Figure 9:**
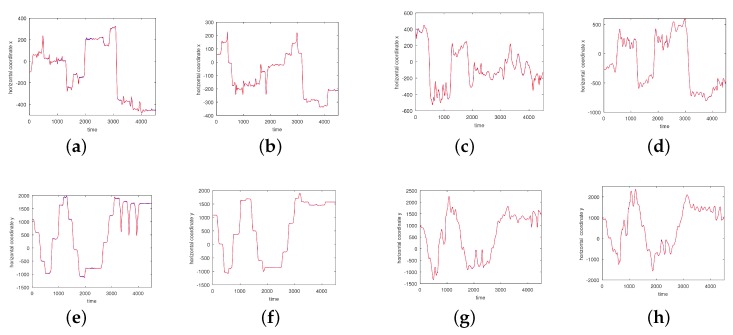
Approximations of *x* (left) and *y* coordinates (right) by 200 neurons and two centers for a simple non-segmented motion (sampled at 120 Hz). The center frequencies are 0.30Hz and 0.72Hz. (**a**) Right heel , *x*-coordinate; (**b**) Left heel, *x*-coordinate; (**c**) Right wrist, distal, *x*-coordinate; (**d**) Left wrist, distal, *x*-coordinate; (**e**) Right heel , *y*-coordinate; (**f**) Left heel, *y*-coordinate; (**g**) Right wrist, distal, *y*-coordinate; (**h**) Left wrist, distal, *y*-coordinate. The red curves show the experimentally observed coordinates and the blue curves give their neural approximations. Both curves coincide up to pixel accuracy in many places.

**Figure 10:**
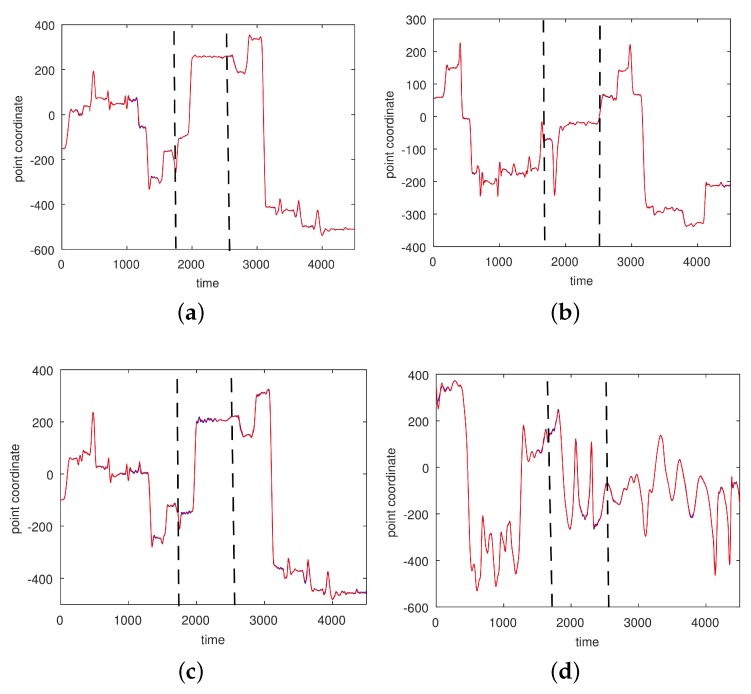
Approximations of *x* coordinates of a complicated motion (CMU 86 Trial 2; sampled at 120 Hz) by a radial basis function (RBF) network with three centers and 100 satellites. The motion was segmented into three intervals [1,1800], [1800,2500], and [2500,4000]. (**a**) Right heel; (**b**) Left heel; (**c**) Right wrist, distal; (**d**) Left wrist, distal.

**Figure 11:**
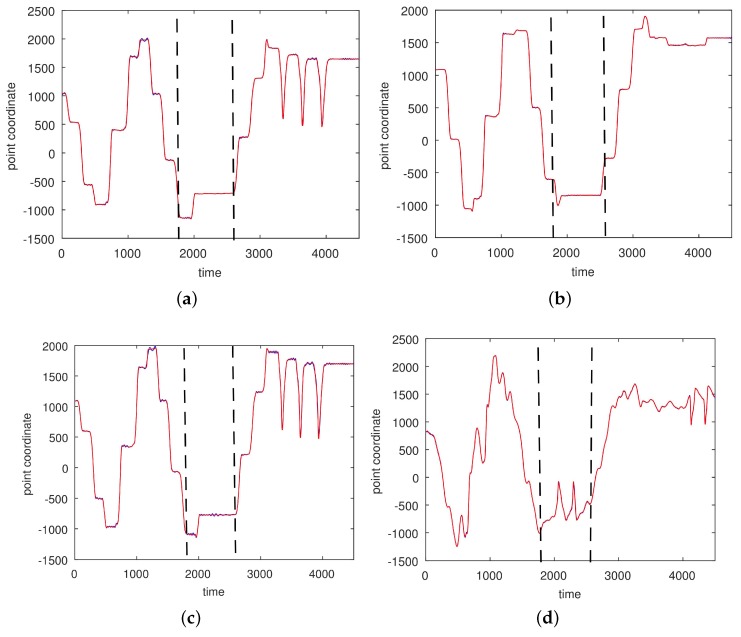
An approximation of *y* coordinates of a complicated motion (CMU 86 Trial 2; sampled at 120 Hz) by an RBF-network with three centers and 100 satellites. The motion was segmented into three intervals [1,1800], [1800,2500], and [2500,4000]. (**a**) Right ankle; (**b**) Left ankle; (**c**) Right heel; (**d**) Left wrist, distal. The red curves show the experimentally observed coordinates and the blue curves represent their neural approximations.

**Figure 12:**
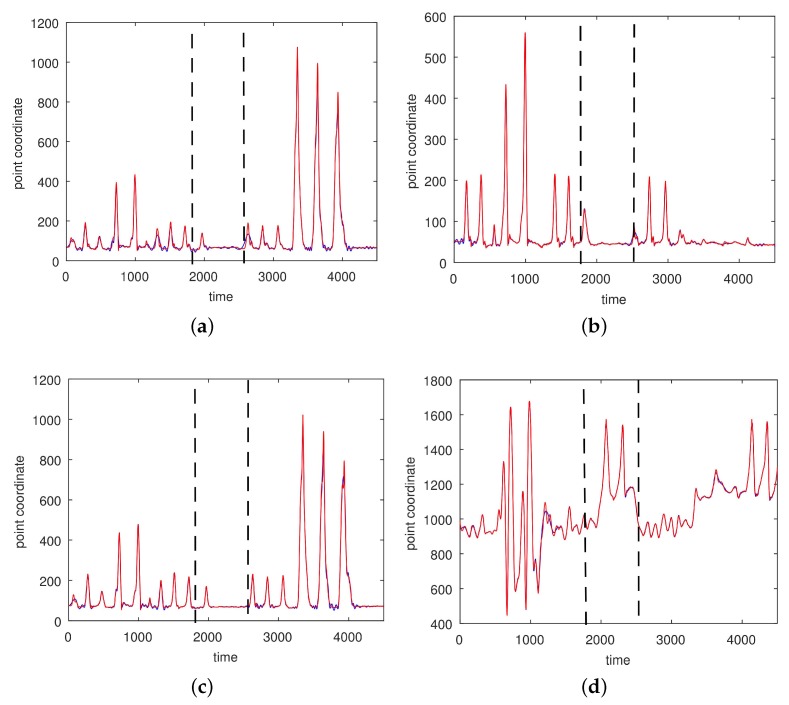
An approximation of the *z* coordinates of a complicated motion (CMU 86 Trial 2; sampled at 120 Hz) by an RBF-network with three centers and 100 satellites. The motion was segmented into three intervals [1,1800], [1800,2500], and [2500,4000]. (**a**) Right ankle; (**b**) Left ankle; (**c**) Right heel; (**d**) Left wrist, distal. The red curves show the experimentally observed coordinates and the blue curves represent their neural approximations.

In Figure 8 and Figure 9 we give the approximations for a simple non-segmented motion. An approximation of a complicated motion (CMU 86 Trial 2) consisting of walking, squatting, running, stretching, jumping, punching, and drinking is given in Figure 10, Figure 11 and Figure 12. For this motion an RBF-network with three centers and 100 satellites was necessary for good approximations on the segmentation of the hand into three parts.

The integral relative accuracies (for *x*, *y*, and *z* coordinates together) are as follows: for the first segment consisting of walking and squats the accuracy is 0.006, for the second segment consisting of running (in a circle) it is 0.002, and for the third one consisting of stretches it is 0.005.

**Figure 13:**
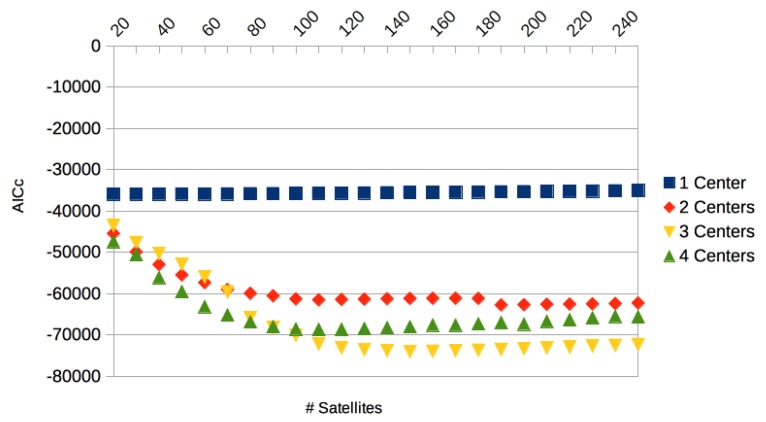
Akaike information criterion corrected for finite sample sizes (AICc) for CMU 86 Trial 1 with 1, 2, 3, and 4 centers and varying numbers of satellites. The global optimum was reached for three centers and 150 satellites.

As the accuracies of course improve when using more satellites, we computed the Akaike information criterion corrected for finite sample sizes (AICc) [42], a likelihood-based measurement for systematic tests involving 20–250 satellites, which weights accuracy against the number of parameters (with lower AIC values being better). The results for Trial 1 with 1, 2, 3, and 4 centers is given in Figure 13.

Notice that only the comparative values are important. The global optimum is reached for three centers and 150 satellites (with a small increase for higher satellite numbers).

### 3.2. Results Based on Algorithmic Segmentations as Pre-Processing Steps

Algorithmic segmentation methods yield much smaller segments than our ad hoc segmentations. Using the method described by Krüger et al. [39] as a pre-processing step, the motion CMU 86 Trial 1 is segmented into six main parts with five transition motions. When taking each of the 11 segments as an input, only 16 satellite neurons per segment are sufficient for good approximations. Notice that the required number of neurons is more than a factor of 15 smaller than the number of frames in each segment.

**Figure 14:**
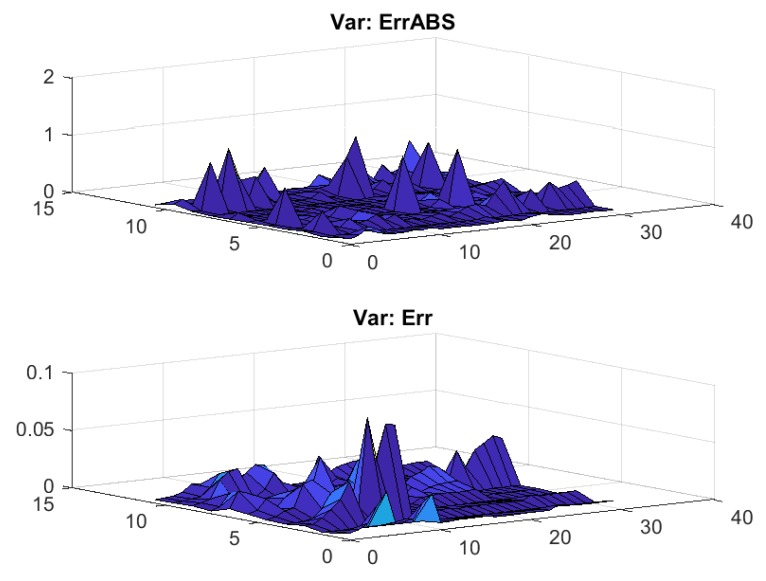
Absolute error (ErrABS) and relative integral errors (Err) for all 31 markers and all 11 segments of CMU 86 Trial 1 using two centers and 49 satellites.

In Figure 14 the absolute and relative integral errors for all 31 markers and all 11 segments of CMU 86 Trial 1 using two centers and 49 satellites are given. The differences between the relative integral errors and the absolute errors can be explained by the large motions of some markers in some segments. We observe a certain ruggedness of the fitting landscape, which can be explained by the rather complicated nature of the motions and the transitions between motions of very different characteristics.

**Figure 15:**
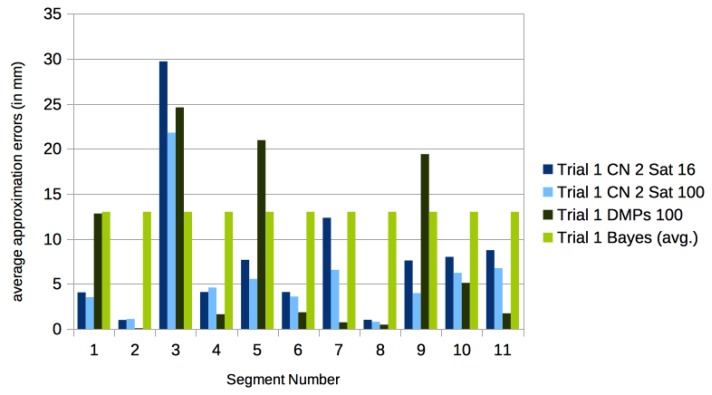
Approximation errors (in mm) for the algorithmically found segments of motions in CMU 86 Trial 1. We give the errors for using 16 and 100 satellites. As a comparison we give the results using rhythmic dynamic movement primitives (DMPs) with 100 basis functions computed with pydmps, and the average approximation error of the Bayesian approach reported in [28] (Table 3). Notice that segments 2, 4, 6, and 8 are short transitional motions between the neighboring segments. The average over all segments is 8.7 mm for the DMPs, 7.9 mm for two centers and 16 satellites, and 6.7 mm for two centers and 100 satellites.

In Figure 15 a systematic comparison of the approximation errors over the different segments in CMU 86 Trial 1 are given when using 16 satellites respectively 100 satellites.

### 3.3. Comparison with Other Approaches

In [28] a method for marker reconstructions based on local similarity searches, building local linear models of found similar motions, and using this information as priors in a pose-wise reconstruction process reported results on marker accuracy on the motions of family CMU 86 from the CMU mocap database. The overall Bayesian framework is similar to that already suggested by [27]. The reported average joint error for CMU 86 Trial 1 is 1.30 cm ([28], Table 3), when taking prior information of motions from the CMU database into account. In our approach the average joint errors are more than one order of magnitude smaller. Although the results are not fully comparable, it is encouraging to see that our approach gives better results even without relying on prior information of other motions, as the Bayesian approach used in [28] does.

**Figure 16:**
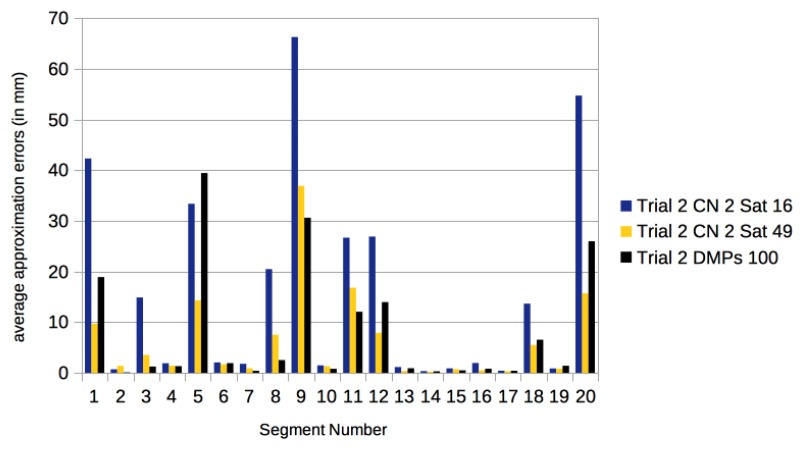
Approximation errors (in mm) for the algorithmically found segments of motions in CMU 86 Trial 2. We give the errors for using 16 satellites and 49 satellites. As a comparison we give the results using rhythmic DMPs with 100 basis functions computed with pydmps. The average over all segments is 8.0 mm for the DMPs, 15.6 mm for two centers and 16 satellites, and 6.3 mm for two centers and 49 satellites.

We approximated the marker trajectories of the segmented motions of CMU motion 86 Trial 1 and Trial 2 with DMPs using the pydmps implementation by Travis DeWolf, which is available at https://github.com/studywolf/pydmps. We have used the code for the rhythmic DMPs (using 100 basis functions) on the algorithmically segmented motions. The results in comparison to our centralized networks are detailed in Figure 15 and Figure 16.

## 4. Discussion and Conclusions

We have shown that marker trajectories of representative body parts can be approximated well by centralized networks consisting of very few centers as oscillators—2 to 3 oscillators have been shown to be sufficient even for rather complicated motions. The needed satellites required even for very good approximations are one to two orders of magnitude smaller than the number of frames considered; hence, our technique yields very compact representations and compresses marker trajectories. The learned frequencies of one marker could be transferred to other markers, so our technique seems to be capable of the motion reconstruction problem from a few markers [27]. As this problem is of particular practical interest if the input data are not marker positions but sensor readings of inertial measurement units [40,41], an application of our method to this setting is of interest. As the accuracy of reconstruction at the level of a single marker is very good, we presume that such a technique could also yield much better reconstruction results than the existing Bayesian approaches. In future work we will investigate this line of research. Additionally, the use of surface electromyography (EMG) has become an increasingly practical sensor technology for human motion interaction (e.g., the Myo Gesture Control Armband), and our technique can be used for sensor data of different kinds. Additionally, for investigating surface EMG signals of animal motions, the centralized networks might yield another basic technique, which we will test on existing data sets [43].

While with the dynamic movement primitives (DMPs) the use of one oscillator has been commonly used and the use of radial basis functions closely correspond to our techniques, we can readily use more than one oscillator. In our experiments, the use of two (or three) oscillators yielded better results than using just one. The idea of switching has also been proposed in the context of DMPs [25]—yielding in some sense a conceptual ad hoc extension. As has been noted in [25] (Section 2.3.5), the modeling easily becomes complex. In our proposal the switching module stays within the realm of centralized networks. In principle, our techniques should be applicable in all contexts in which DMPs have been used, yielding a simpler modeling alternative.

The oscillator frequencies give very useful semantic information on motions that should be widely applicable. By searching for similar vectors of oscillator frequencies, our technique can also give a basis for motion retrieval, which in contrast to other techniques does not involve similarity measures for poses first [28] but works directly on marker trajectories. As vectors of oscillation frequencies are readily indexable, efficient retrieval from even huge motion databases is possible—and fine tuning the query regarding the weighting of different body parts is even possible without a re-indexing of the entire database. By manipulating the oscillator frequencies or transferring them to other marker positions, the presented techniques are also capable of various motion adaption and synthesis tasks, which range from a new technical basis for the classic ideas by Pullen and Bregler [44] to ideas related to motion fields [45]. It will be the topic of future work to explore these directions in more detail.

In our current method there is no need to use a priori knowledge on human motions by referring to similar known motions, as is the basis of Bayesian approaches [26,27,28]. Being an advantage on the one hand, it is on the other hand a disadvantage if such a priori knowledge on “similar motions” is available. Incorporating such a possibility is very much in the realm of neural networks, and will be a topic for future research.

Moreover, centralized networks should also be applicable in the context of motion anticipation: by extrapolation from the past into the future, the presented technique also has the potential for full body motion anticipation in the short-term when staying within a fixed tuple of oscillator frequencies and for the mid-term range when using switching. We will explore this possibility within our future research in the collaborative research unit “Anticipating Human Behavior”, funded by *Deutsche Forschungsgemeinschaft* under grant number FOR 2535. 

## Figures and Tables

**Table 1:** Integral relative accuracies of approximations by centralized networks of CMU 86 Trial 1 using different numbers of oscillators and satellites. The motion was segmented into four segments at frames [1,1300,2000,3000,4500].

Number of Oscillators	Number of Satellites	Integral Relative Accuracies			
		Segment 1	Segment 2	Segment 3	Segment 4
1	100	0.2133	0.0827	0.423	0.0749
2	25	0.1207	0.0486	0.0405	0.076
2	50	0.0351	0.0089	0.0084	0.0586
2	100	0.0189	0.0068	0.0029	0.0299
3	25	0.0832	0.0253	0.0297	0.0685
3	100	0.0133	0.0063	0.0031	0.0071
